# Linoleic Acid-Based Transferosomes for Topical Ocular Delivery of Cyclosporine A

**DOI:** 10.3390/pharmaceutics14081695

**Published:** 2022-08-15

**Authors:** Onyinye Uwaezuoke, Lisa C. Du Toit, Pradeep Kumar, Naseer Ally, Yahya E. Choonara

**Affiliations:** 1 Wits Advanced Drug Delivery Platform Research Unit, Department of Pharmacy and Pharmacology, School of Therapeutic Sciences, Faculty of Health Sciences, University of the Witwatersrand, 7 York Road, Parktown, Johannesburg 2193, South Africa; 2 Department of Neurosciences, Division of Ophthalmology, University of the Witwatersrand, 7 York Road, Parktown, Johannesburg 2193, South Africa

**Keywords:** topical ocular drug delivery, transferosomes, linoleic acid, cyclosporine A, nanoparticle drug-delivery systems

## Abstract

Delivering high-molecular-weight hydrophobic peptides, such as cyclosporine A, across the corneal epithelium remains a challenge that is complicated by other physio-anatomical ocular structures that limit the ocular bioavailability of such peptides. Transferosomes have previously been used to improve transdermal permeability, and have the potential for improving the ocular corneal permeability of applicable drugs. In this study, transferosomes for the potential ocular delivery of cyclosporine A were investigated. Linoleic acid was evaluated for its effect on the stability of the transferosomes and was substituted for a portion of the cholesterol in the vesicles. Additionally, Span^®^ 80 and Tween^®^ 80 were evaluated for their effect on transferosome flexibility and toxicity to ocular cells as edge activators. Attenuated Total Reflectance–Fourier Transform Infrared spectroscopy (ATF-FTIR), differential scanning calorimetry (DSC), and dynamic light scattering (DLS) were used to evaluate the physicochemical parameters of the blank and the cyclosporine A-loaded transferosomes. Cyclosporine A release and corneal permeability were studied in vitro and in a New Zealand albino rabbit corneal model, respectively. The linoleic acid contributed to improved stability and the nano-size of the transferosomes. The Tween^®^-based formulation was preferred on the basis of a more favorable toxicity profile, as the difference in their corneal permeability was not significant. There was an initial burst release of cyclosporine A in the first 24 h that plateaued over one week. The Tween^®^-based formulation had a flux of 0.78 µg/cm^2^/h. The prepared transferosomes demonstrated biocompatibility in the ocular cell line, adequately encapsulated cyclosporine A, ensured the corneal permeability of the enclosed drug, and were stable over the period of investigation of 4 months at −20 °C.

## 1. Introduction

Topical ocular delivery is fraught with numerous challenges, arising mainly from the physio-anatomical barriers posed by ocular structures in their normal physiological line of duty [1]. A formidable barrier to topically applied substances is regularly presented by the corneal epithelium, barely permitting the passage of low molecular weight substances by diffusion [2]. The corneal epithelial cells allow the passage of lipophilic drugs that then is barred by the corneal stroma, which is hydrophilic [3]. In addition, the paracellular passage of substances is heavily challenged by the tight junctions presented by the cornea [4]. Consequently, the drive to deliver drugs across the cornea via topical ocular formulations is a continuing venture. There is also the additional challenge of increasing the time that such formulations stay on the ocular surface. This surface residence time is shortened by the blink reflex, as well as the constant flow of the tear film. Various colloidal systems, such as polymeric micelles [5], nanoparticles [6] nanocapsules [7], microemulsions [8] and vesicular systems, have been explored to overcome many of these challenges. Among these, vesicular systems such as liposomes have stood out, overcoming the polarity issues associated with many of the new chemical entities (and which limit their passage through many biological membranes) and bringing biocompatibility, extended release, reduced systemic side-effects, and improved ocular biodistribution to topical ocular delivery [9].

Many of the ocular conditions require the topical application of medicaments for their treatment. Among these, the most frequently occurring are dry eye syndrome (keratoconjunctivitis sicca), keratoconus, and keratitis [10]. Cyclosporine A (CysA) is a strong immune suppressive oligopeptide with 11 amino acid residues that has found use in many inflammatory conditions of the eye, such as non-infectious uveitis and vernal keratoconjunctivitis corneal healing [11]. Even though the systemic administration of CysA, which was the initial mode of administration for ocular interventions, achieves high concentrations in ocular tissues, the high incidence and level of side effects arising from such a systemic administration is driving the search for topical formulations that could achieve the same therapeutic concentrations in the ocular tissues [12]. Unfortunately, the high molecular weight (1202.6 Da), strong hydrophobicity (Log P lies between 1.4 and 3 depending on the solvent), and the presence of formidable barriers leaves little room for flexibility in terms of formulation maneuvers to achieve the optimum availability in ocular tissues from topical applications [11,13]. Nevertheless, various nanoplatforms have been explored for overcoming some of these issues with the physicochemical properties of CysA. Many of these platforms utilized excipients, to enable the solubilization and stabilization of CysA, some of which may be contraindicated in the disease conditions which these formulations are intended to treat, as a result of the additional disruptions brought about on the corneal surface [5,14]. Allergan Inc., USA (now AbbVie) was the first company to push a nanoemulsion formulation of CysA to the market. Most of the other marketed CysA topical drops are micellar solutions in which various surfactants, alcohols, co-solvents, and even some excipients that have been shown to be harmful to ocular health, were used. One such excipient is EDTA, which is employed for the solubilization of CysA [4,15].

Transferosomes were introduced three decades ago to further expound the capabilities of the liposomal systems though directed to improve the delivery via the stratum corneum of the skin [16]. Transferosomes as ultra-elastic lipid vesicles have, since inception, been exploited in transdermal delivery for their exceptional permeability and the deformability properties imparted to the bilayer of regular vesicles, due to the presence of edge activators, such as Tween^®^ 80, Span^®^ 80, and sodium cholate [17]. Transferosomes are currently being deployed to improve the permeability of the stratum corneum to a variety of drugs. The success of transferosomes as a transdermal delivery system has been attributed partly to the osmotic gradient that exists across the outer and inner skin layers, and partly to the ability of transferosomes to deform while passing pores that are much smaller than them. This understanding and the existence of similar gradients across the corneal epithelium and stroma suggest that the same possibilities may exist for the permeation of large molecules across the tight junctions of the eye cornea. One major challenge that may deter the use of transferosomes in the corneal drug delivery has to do with the possible toxicity of the surfactants employed as the edge activators to ocular cells and therefore, requires a careful selection of, and delicate balance between, the edge activators and their use level. A major challenge in dry eye disease is the instability in the tear film as a result of sub-optimal tear volume and function [10]. While CysA has a strong stimulating effect on tear fluid production, the excipients that will adequately stabilize the produced tear fluid are still lacking in many commercial formulations. As a result of these considerations, linoleic acid was introduced as a vesicle component to complement the pharmacological action of cyclosporine A in the treatment of the targeted ocular condition. Linoleic acid is an essential fatty acid that has been shown to stabilize the tear film when applied topically [18], and could therefore contribute to the therapeutic experience in patients with dry eye syndrome, in whom tear film stability is a major challenge contributing to the discomfort from the disease [19]. Daull and co-workers [13] had earlier concluded, after reviewing many formulations of cyclosporine A currently on the market, that the vehicle carrier in cyclosporine A topical formulations could contribute to a significantly improved performance.

The benefits of transferosomes that have chronicled its application in transdermal delivery underscore their potential in topical ocular delivery. Thus far, no study has explored the possibility of transferosomes in ocular applications; hence, in this investigation, a topical transferosomal formulation was proposed and explored as an alternative topical formulation for CysA, particularly in the treatment of dry eye disease. In this investigation, transferosomes, which are generally composed of phospholipids and an edge activator as a membrane-softening agent, were formulated. Both Tween^®^ 80 and Span^®^ 80 were selected for investigation, based on their demonstrated efficacy in facilitating the deformability of the transferosomes. The additional challenge of tear film stability that accompanies and complicates ocular conditions, such as dry eye, informed the decision to include linoleic acid in the formulation. Linoleic acid has been found to have a stabilizing effect on tear film. In addition, linoleic acid can contribute to permeation enhancement as a fatty acid since other fatty acids, such as oleic acid, have demonstrated potential as permeation enhancers in dermal formulations [20]. Additionally, linoleic acid was explored for its effect on stabilizing the lipid bilayer of the transferosome, thereby acting as both an excipient and an active ingredient. Spectroscopic methods were used to characterize and confirm the inclusion of CysA in the form of transferosomes. Further physicochemical parameters, such as size and zeta potential, were studied, as well as the biocompatibility in an ocular cell line. In addition, the ex vivo corneal permeability of the developed transferosomes were assessed, employing rabbit corneal tissue.

## 2. Materials and Methods

### 2.1. Materials

The cyclosporine A was purchased from LEAP pharma (DLD Scientific, South Africa). The Tween^®^ 80, Span^®^ 80, linoleic acid, soy phosphatidylcholine, cholesterol, and the MTT assay kit were purchased from Sigma-Aldrich (St. Louis, MO, USA). The ultrapure water (Milli Q, water, Sigma-Aldrich, Burlington, MA, USA) was used. All of the other solvents were obtained and used without further purification.

### 2.2. Preparation of the Transferosomes

**Scheme 1:**
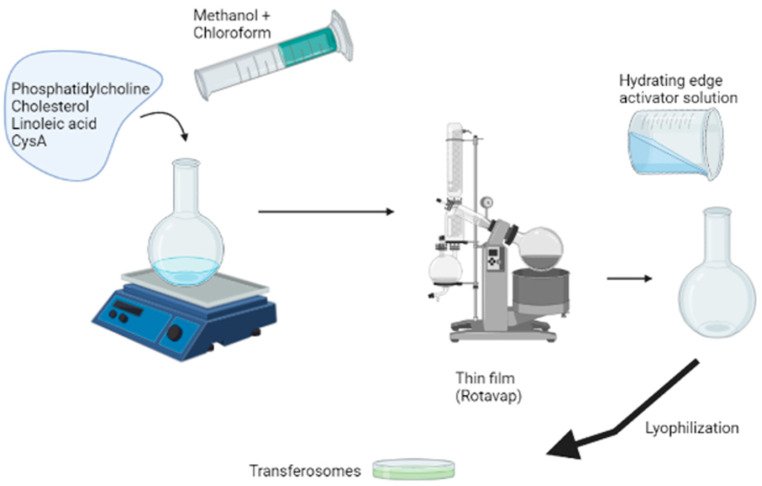
Formulation of the transferosomes.

The thin film method was employed in the preparation of blank and CysA-loaded transferosomes, as depicted in Scheme 1 using the formulation variables outlined in Table 1. The lipids, cholesterol, and linoleic acid were dissolved in an appropriate volume of chloroform–methanol solution, mixed in a ratio of 3:1. For the CysA-loaded transferosome, the cyclosporine was added in the film-forming solution. The resulting solution was evaporated in a rotary shaker (Rotavapor1 RII, Büchi Labortechnik AG, Flawil, Switzerland) to obtain a thin film which was subsequently hydrated using either a Tween^®^ or Span^®^ solution constituted in artificial tear fluid (ATF) of pH 7.4. The concentration of the Tween^®^ or Span^®^ solution was varied, as shown in Table 1 and incorporated either as part of the hydrating fluid or as part of the lipid film. The resulting multilamellar vesicles were ultrasonicated for 5 min in ice using a probe sonicator (Sonics Vibra cell, Newtown, CT, USA) set at a 20 s on and 5 s off cycle, and an amplitude of 50%. The obtained nanovesicles were subsequently lyophilized (Freezone 12 freeze drier, Labonco, Kansas City, KS, USA) and characterized appropriately. A 2% sucrose solution was used as a cryo-protectant. The effect of the linoleic acid inclusion on the physicochemical characteristics of the transferosomes was also evaluated by preparing Tween^®^ 80-based transferosomes, with and without linoleic acid.

### 2.3. Characterization of the Transferosomes

The size, zeta potential, and polydispersity index of the prepared transferosomes were determined in triplicate, using dynamic light scattering (DLS) on the ZetaSizer NanoZS (Malvern Instruments, Malvern, UK). These measurements were undertaken shortly after sonication and after 4 months of storage at −20 °C in a freezer (GL I472QPZX, LG Korea) [21].

A FEI Tecnai T12 transmission electron microscope (TEM, Hillsboro, OR, USA) and the ZEISS Sigma 300 VP with a ZEISS ‘smart SEM’ software (Field Emission Scanning Electron Microscope, SEM, FEI, Orlando, FL, USA) were used to characterize the morphology of the transferosomes. A dispersion of the lyophilized transferosomes was prepared in deionized water to a concentration of 0.5 mg/mL and dropped on a copper grid. The excess was dabbed with a filter paper and the dispersion allowed to dry. The grid was subsequently covered by a drop of 2% *v*/*v* phosphotungstic acid and imaged with the FEI Tecnai T12 transmission electron microscope. For the SEM imaging, a drop of the dispersion was dropped onto a two-sided carbon tape mounted on an aluminum stub. The dispersion was dried, and sputter-coated with gold/palladium before imaging.

Differential scanning calorimetry (DSC) was undertaken (Mettler Toledo, DSC, STARe System, Swchwerzenback, ZH, Switzerland) to study the heat transitions in both the CysA-loaded and unloaded vesicles.

Attenuated Total Reflectance–Fourier Transform Infrared spectroscopy was undertaken on a Perkin Elmer Spectrum 2000 ATR-FTIR spectrometer (PerkinElmer 100, Llantrisant, Wales, UK), fitted with a single-reflection diamond. A MIRTGS detector was used to study the surface transitions and functional groups on both the cyclosporine A-loaded and unloaded transferosomes.

The stability of the transferosomes was assessed by storing lyophilized transferosomes at a representative storage temperature of −20 °C over a period of 4 months. At specified time intervals, the size distribution, polydispersity index, and zeta potential of the samples were measured.

The elasticity of the transferosomes was studied, using a modified method as applied by Jain and coworkers [22]. An appropriate amount of the lyophilized transferosomes was dispersed in artificial tear fluid (ATF) to formulate a stock suspension that was diluted appropriately before analysis. The stock suspension (one part) was diluted with nine parts of ATF and the particle size determined. Subsequently, 1 mL of the diluted system was extruded through a 100 nm polycarbonate filter. The volume extruded was noted, as well as the hydrodynamic size of the extruded dispersion. The extrusion process was repeated five times.

### 2.4. Encapsulation Efficiency and In Vitro Drug Release from Cyclosporine A-Loaded Transferosomes

The direct method was employed in determining the quantity of the drug encapsulated in the transferosomes [23]. To this end, a dispersion of the lyophilized drug-loaded transferosomes was prepared in ATF and the absorbance read at a wavelength of 207 nm on an IMPLEN NanoPhotometer^®^, (Implen GmbH, Munchen, Germany) The concentration was subsequently determined from a standard calibration curve of cyclosporine A, prepared using methanol: ATF (9:1) solution as solvent. The encapsulation efficiency (EE%) was subsequently calculated, using Equation (1):

(1) $$\mathrm{EE}\%=\frac{Actual\;amount\;of\;drug\;entrapped\;in\;the\;transferosome}{Amount\;of\;drug\;incoporated\;in\;the\;transferosome}\times 100$$

In a similar process, the drug release from the transferosomes was determined, using a modified USP dissolution apparatus, commonly applied as an in vitro drug release test for colloidal drug carriers [24]. Briefly, one end of a glass tube (open at both ends) was covered with dialysis tubing (12,000–14,000 MWCO), which had initially been equilibrated in ATF at 37 °C. The prepared transferosome dispersion in ATF (1 mL) was introduced from the other open end and suspended in 100 mL of ATF of pH 7.4 as the dissolution medium. The dissolution medium was stirred at 50 rpm and maintained at 37 °C ± 1 °C. At scheduled time intervals, a 1 mL sample was withdrawn from the dissolution medium and replaced with an equivalent volume of ATF and the drug content subsequently calculated from the absorbance reading acquired from the Implen NanoPhotometer™ (Implen GmbH, Munchen Germany).

### 2.5. Ex Vivo Corneal Permeability of the Transferosomes

The corneal permeability of the formulations was determined, using the cornea from New Zealand albino rabbits. The rabbits were selected because the size of the rabbits’ cornea is similar to that of humans. The eyes of the rabbits were enucleated, and the cornea removed accompanied by about 3 mm of scleral tissue. These were immediately rinsed with ATF and wrapped with film to prevent dehydration. Subsequently, they were stored at 4 °C until used within 4 h of harvest [4,25]. The permeability study was undertaken in a Franz diffusion cell (PermeGear Inc., Bethlehem, PA, USA) with three cells. The cornea was used as the separating membrane between the donor and receptor chamber (enclosed in silicon rings) with the epithelial surface facing upward to the donor chamber. The receptor chamber was filled with 12 mL of degassed ATF, stirred at 60 rpm, and the system temperature maintained at 36 °C by means of a circulating water bath. A transferosomal dispersion in ATF was made and 1 mL introduced into the donor chamber. At 3, 6, and 24 h, 0.4 mL was withdrawn from the receptor chamber and replaced with an equal volume of degassed ATF. These were subsequently analyzed and the amount of drug that had passed through was determined from the cyclosporine A calibration curve.

### 2.6. Cytotoxicity Studies of the Transferosomes in Human Retinal Epithelial Primary Cell Line

The MTT, (3-[4,5-dimethylthiazol-2-yl]-2,5-diphenyltetrazolium bromide), assay to determine the cytotoxicity of the transferosomes was carried out using an ocular cell line, human retinal epithelial primary (HREP) cells. The HREP cells were grown in DMEM: HAM’s F12 (50:50) medium supplemented with 10% FBS and 1% penicillin/streptomycin. The cells were grown until they were 80 to 90% (about 48 h incubation) confluent in an incubator set at a temperature of 37 °C with 5% CO_2_. Thereafter, the cells were detached using 2 mL of 1% trypsin. The cells were thereafter seeded in two 96-well plates at a cell density of 40,000 cells per plate for the HREP cells and further incubated for 24 h. The plates were thereafter treated with 10 µL of a 5 mg/mL transferosome dispersion. After the treatment, the plates were incubated at a temperature of 37 °C with 5% CO_2_. At 24 and 48 h, respectively, each well plate was treated with 10 µL of (3-[4,5-dimethylthiazol-2-yl]-2,5-diphenyltetrazolium bromide) (MTT) at a final concentration of 5 mg/mL. Each plate was further incubated for 4 h in a humidified atmosphere at 37 °C and 5–6% CO_2_. A solubilization reagent (100 µL) was then added and the plate allowed to stand overnight in the incubator. The absorbance of the developed purple formazan crystals was then read in a VX3 microplate reader at 570 nm. The percent viability of the cells was subsequently calculated using Equation (2):

(2) $$\%\;Viability=\frac{Absorbance\;of\;treated\;well-absorbance\;of\;blank}{Absorbance\;of\;control-absorbance\;of\;blank}\times 100$$

The ATF was used as the negative control, the 5-fluorouracil and DMSO were used as the positive controls, and the untreated wells were used as the blank.

### 2.7. Statistical Analysis

The continuous variables with a normal distribution, such as particle size, size distribution, zeta potential, etc., are reported as mean ± SD. Comparisons to establish the statistical significance where needed were undertaken using one way ANOVA and Student’s *t*-test at *p* = 0.05. All of the statistical analyses were performed using Microsoft Excel 2016 (Microsoft Corp., Redmond, WA, USA) and GraphPad by Dotmatics (Boston, MA, USA).

## 3. Results

### 3.1. Formulation and Characterization of the Transferosomes

The hydrodynamic diameter, polydispersity index, and zeta potential transferosomes prepared using 1% *v*/*v* Span^®^ 80 or Tween^®^ 80, together with linoleic acid, are displayed in Table 2. During preliminary studies, the concentration of the surfactants employed was varied between 1% *v*/*v* and 2% *v*/*v* to establish a compromise between toxicity, and elasticity/deformability. A concentration of 1% *v*/*v* of Span^®^ 80 or Tween^®^ 80 was employed in the ensuing investigations, based on the ability to form stable, deformable transferosomes of adequately low toxicity. The results displayed in Table 2 indicate that the hydrodynamic diameter of the transferosomes formed using the Span 80^®^ was higher than that formed by the Tween 80^®^. Contrary to the norm, loading CysA seemed to yield smaller sized transferosomes. Most of the studies report an increase in the size of vesicles after drug loading.

Lyophilization had a potentially stabilizing effect on all of the vesicles. This is evident from the results of the zeta potential, which depict the magnitude of charges that tend to cause repulsion between two vesicles in close proximity. The values above +30 mV and below −30 mV are considered acceptable for the stability of the colloids [26]. Even though the zeta potentials for all of the formulations were initially within an acceptable range, they all somewhat increased after lyophilization. The nanovesicles, by default, are prone to membrane destabilization that result from the double effect of environmental factors, such as moisture, oxygen, and the presence of large quantities of unsaturated fatty acids in an aqueous environment [27]. Lyophilization, which is often employed as one of the methods to limit this form of instability, can fracture the delicate vesicle membrane as a result of the effect of ice crystals. The stabilization can, therefore, be the result of an intricate interplay between the concentration and type of cryoprotectants used and the fracturing effect of lyophilization [28]. For Tween^®^ 1% formulations, the stabilization effect is noted in the form of a favorable shift from a value of −18.9 ± 1.6 to −24.43 ± 2.88 for the CysA-loaded formulation.

To study the effect of linoleic acid, the preliminary formulations were prepared with and without linoleic acid. In all of the formulations studied, the zeta potential and, thus, the stability to aggregation was improved by the addition of linoleic acid both for the CysA-loaded and blank Tween^®^ 80 transferosomes. The size of the transferosomes was also comparatively reduced. The results from a representative lyophilized Tween^®^ 80 1% *v*/*v* CysA-loaded formulation are shown in Table 3. The two-tailed *p*-value for size and the zeta potential comparison between the representative formulations determined by GraphPad were less than 0.0001 and 0.0002, respectively, thus notably significant.

### 3.2. Physicochemical Characterization of the Transferosomes

**Figure 1:**
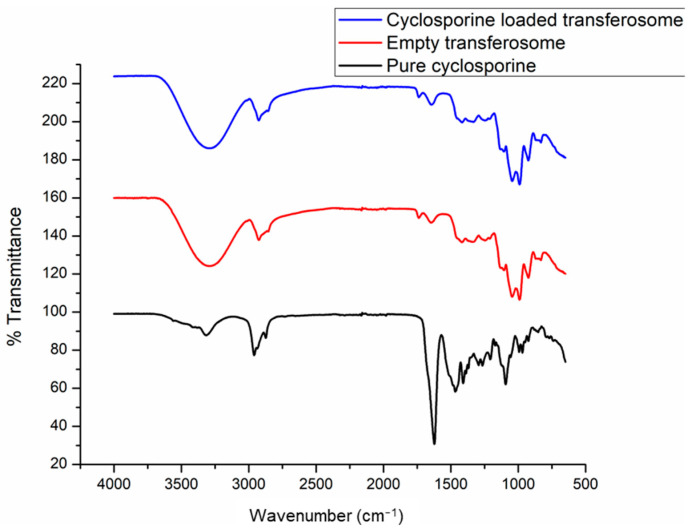
FTIR analysis of drug-free and drug-loaded transferosomes.

Fourier-transform infrared spectroscopy is usually used to study and characterize the nature of functional groups occurring at molecular surfaces. The results displayed in Figure 1 depict the FTIR fingerprints for pure CysA, blank transferosomes, and CysA-loaded transferosomes. The FTIR of both the blank and CysA-loaded transferosomes are identical, showing the same band intensities for identified functional groups occurring at the same wave numbers. None of the characteristic bands for CysA could be detected in the CysA-loaded transferosomes. This confirms the encapsulation of CysA within the transferosome vesicles and that no new bonds were formed when CysA was loaded into the transferosomes [29].

**Figure 2:**
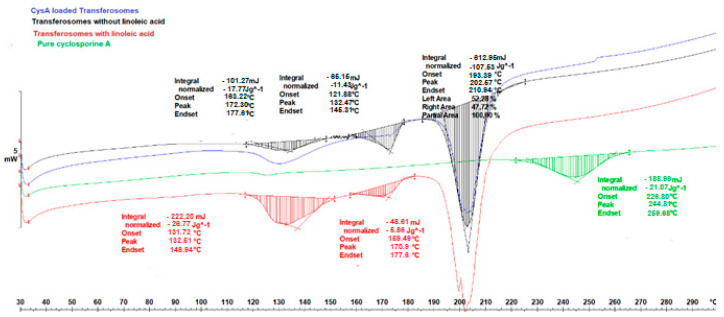
Differential scanning calorimetry of pure CysA, drug-free (with and without linoleic acid), and CysA-loaded transferosomes.

Figure 2 depicts the thermograms derived from the differential scanning calorimetric analysis performed on pure CysA, the unloaded transferosomes prepared with and without linoleic acid, and the transferosomes loaded with CysA. DSC was applied to characterize the thermal transitions between phases that occur in materials, resulting from temperature changes as directed by heat flow. The thermogram of bilayers of pure lipids generally depicts the transition temperature at which the lipid transforms from gel to a liquid crystalline phase [30].

In Figure 2 the thermal transitions occurring in the lipid vesicles prepared in this study are evident. These vesicles were cryo-protected with a 2% sucrose solution before being lyophilized. The exothermic peaks that occurred at ~132 °C in all of the transferosomes represented the glass transition temperature. In the melting peaks occurring at 170.97 °C and 172.50 °C, respectively, for the transferosomes with and without linoleic acid; there was a slight depression in the melting temperature with respect to the transferosomes without linoleic acid. Londoño and co-workers [31] studied the thermal transitions in soy phosphatidylcholine-based ethosomes and observed peaks at 187.5 °C, which they attributed to the interdigitation of lipid chains that represents the presence of crystalline structures through which the heat flux is constant. The peaks, observed between 170.97 °C and 172.50 °C for the vesicles in the current study, can thus be attributed to such interdigitation of the vesicle chains. The melting endotherm for pure CysA in this study was found at ~130 °C (the melting point is reported as 148–151 °C [32]) and the decomposition started at 226.80 °C. These peaks, however, disappeared completely from the thermogram of the CysA-loaded transferosome, thus indicating the efficacy and stability of the encapsulation process. Similar observations were previously reported [29].

**Figure 3:**
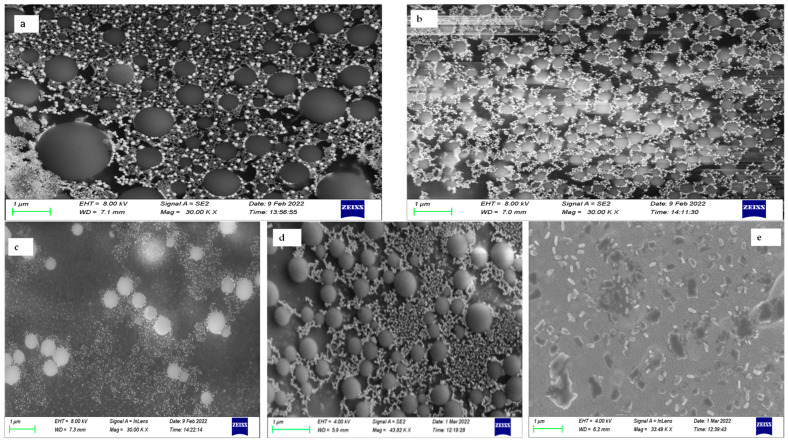
SEM images of transferosomes reflecting the impact of drug loading on the polydispersity and size for. (**a**) blank Span^®^ transferosomes; (**b**) CysA-loaded Span^®^ transferosomes; (**c**) blank Tween^®^ transferosomes; (**d**) CysA-loaded Tween^®^ transferosomes; and (**e**) lyophilized Tween^®^ transferosomes. Scale bar represents 1 µm.

The representative SEM images shown in Figure 3 confirm the poly-dispersed nature of the prepared transferosomes obtained during DLS size analysis. The approach employed for the incorporation of the edge activators was explored for the possible effects on the morphology of the transferosomes. The morphology of the transferosomes prepared with Span^®^ 80 as part of the hydrating fluid is shown in Figure 3a. The stabilizing effect of CysA loading into the transferosomes (Figure 3b) is noted as more uniformly distributed spheres of smaller diameters when compared with the blank. This effect corroborated the reduction in the hydrodynamic size with CysA loading, observed following the DLS size analysis. While Figure 3c represents the transferosomes prepared via the incorporation of Tween^®^ 80 as a constituent of the hydration fluid, Figure 3d depicts the morphology of the CysA-loaded Tween^®^ 80 transferosomes, resulting from incorporating the edge activator, Tween^®^ 80, into the film-forming solution. Apart from a visually imperceptible difference in the size, the morphology is the same. This indicates that the method of incorporation of the edge activator had a minimal impact on the size and size distribution. A representative image of the morphology of the lyophilized samples is depicted in Figure 3e. The effect of lyophilization and the cryoprotectant used were obvious, as the spheres developed an elongated shape. This morphology was also observed in the TEM images obtained for the lyophilized samples prepared with Tween^®^ 80.

**Figure 4:**
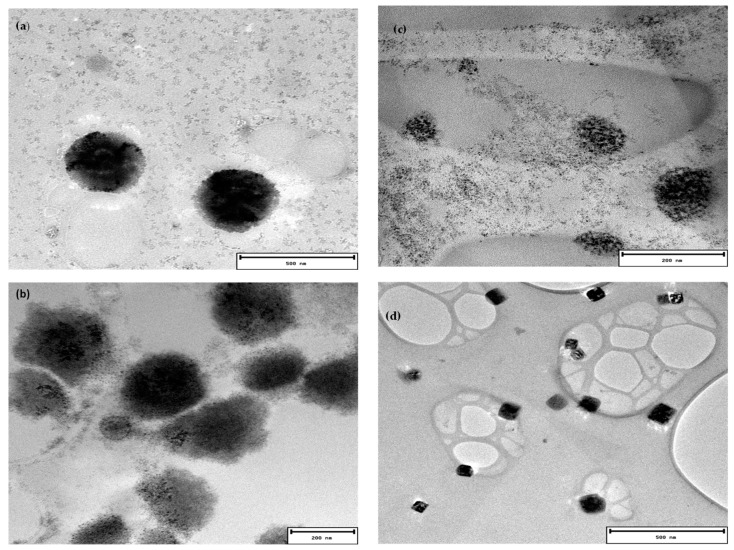
TEM images of lyophilized (**a**) blank Span 80^®^ transferosomes; (**b**) CysA-loaded Span 80^®^; (**c**) blank Tween 80^®^ transferosomes; and (**d**) un-lyophilized Tween 80^®^ transferosomes. The scale bars for (**a**,**d**) are 500 nm, while those for (**b**,**c**) are 200 nm.

The TEM images depicted in Figure 4 were obtained from the lyophilized samples reconstituted in PBS and dried on a copper grid. The images highlight the spherical nature of the transferosomes. The mean sizes, determined from an analysis of the TEM images for the drug-loaded Span^®^ 80 transferosomes, were about 200 nm, while the sizes of the blanks were in the range of 300 to 400 nm. The TEM-determined sizes for the blank Tween^®^ 80 transferosomes were between 100 and 150 nm for the lyophilized samples, while that of the drug-loaded un-lyophilized sample was in the range of 100 nm. The TEM images in Figure 4 corroborate the morphology observed from the SEM. The samples also showed a somewhat elongated morphology approaching cuboids for the non-lyophilized Tween^®^ samples, as observed in the SEM images.

Table 4 shows the results of the stability evaluations obtained from the representative Tween^®^-based transferosome samples. The differences in the size, size distribution, and zeta potential were statistically different, but all in favor of stability. Even though the average hydrodynamic size of the transferosomes was smaller at the beginning, the polydispersity index was high, showing a high variability in the size distribution. The presence of polydisperse vesicles could have resulted from the method of vesicle formation, since the thin film method is known for the production of multilamellar vesicles [26].

This trend was later reversed, however, evident in the presence of more uniformly distributed larger spheres, that could be due to the effect of annealing that results from the reassembly into bigger vesicles following the disruption in the lamellar membrane by the ice crystals during freezing [33]. The contributions of drug loading towards stability were also observed in terms of increased zeta potential values (−19.07 ± 1.22 mV) when compared to the blank vesicles. This corresponds with previous investigations involving lipid nanocapsules [34].

The results of the flexibility studies undertaken with both blank and CysA-loaded transferosomes are shown in Table 5. Jain and coworkers [22] had previously established the inverse relationship that exists between the volume reduction after extrusion and flexibility. Even though the percentage reduction in size for the drug-loaded vesicles was high, these results represent some degree of fluidity when the molecular size cut-off of the polycarbonate membrane (100 µm) used in the extrusion is considered. Statistically significant differences were obtained (*p* < 0.05) when the means of the percent decrease in the size of the blank and drug-loaded vesicles were compared, indicating that the CysA-loaded vesicles were in a more fluid state than the blank vesicles for both of the surfactants studied. The size and stability of the lipid vesicles were shown to depend on membrane packing, and this in turn depends on the interaction of the proteins and peptide molecules with the lipid bilayer [24]. Likewise, the blank vesicles based on Tween^®^ 80 were more deformable than those based on Span^®^ 80 for the blank vesicles based on consideration of the percent decrease in the size of vesicles. This observation is further confirmed by the volume reduction result, which showed a statistically insignificant difference. On the other hand, the difference in size reduction between the CysA-loaded transferosomes based on Tween 80^®^ and those based on Span 80^®^ were statistically not significant. This observation may have serious implications for the contributions of the drug to the membrane stability and fluidity, as was also observed from the dynamic light scattering experiments.

### 3.3. Ocular Cytocompatibility of Transferosomes

**Figure 5:**
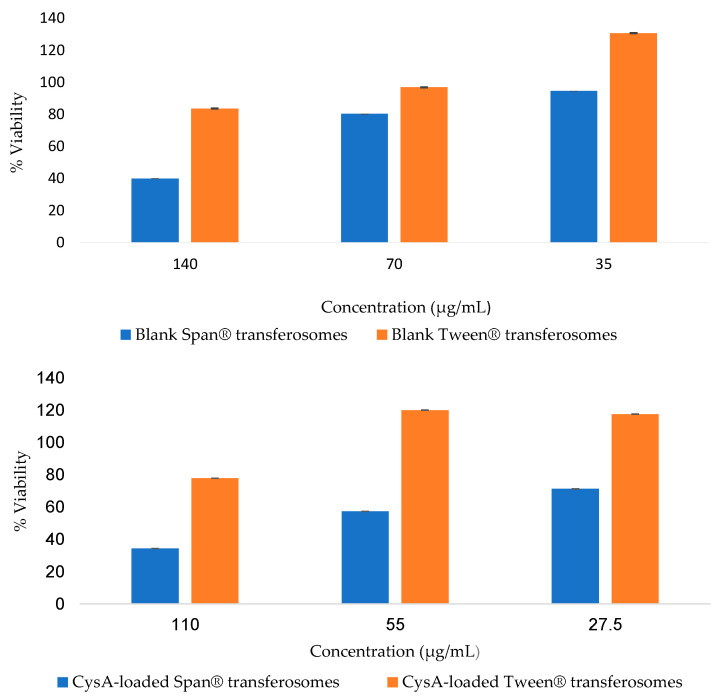
Viability of HREP cells against CysA-loaded transferosomes and blank transferosomes.

**Figure 6:**
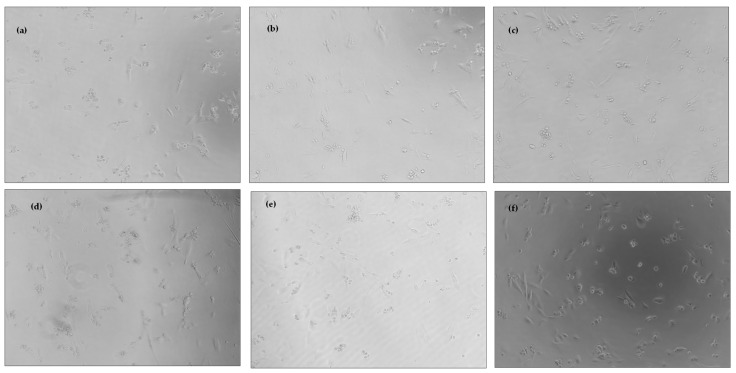
Morphology of HREP cells treated with (**a**) CysA-loaded Span^®^ transferosomes; (**b**) blank Span^®^ transferosomes; (**c**) blank Tween^®^ transferosomes; (**d**) CysA-loaded Tween^®^ transferosomes; (**e**) ATF; and (**f**) before treatment.

In order to evaluate the safety of use of the prepared transferosomes in the eye, a toxicity assay was undertaken in a human retinal epithelial primary cell line (HREP). This cell line was selected because of the very high sensitivity of retinal epithelial cells to exogenous substances. The viability of the cells tested with 3.5 mg/mL stock dispersions of the transferosomes hydrated with surfactant concentrations of 1% *v*/*v* were similar to the viability of the negative control, as displayed in Figure 5. The % viability of the Tween^®^-based transferosomes was higher than the % viability of the Span^®^-based transferosomes at all of the concentrations tested. The viability of the HREP cells to the blank transferosomes was, likewise, similar to the viability of the CysA-loaded transferosomes. Figure 6 shows the morphology of the HREP cells treated with the dispersions of the Span^®^ 80 and Tween^®^ 80-based transferosomes. The morphology of the cells reflects the environment in which the cells are growing. The abnormalities in the morphology can thus be a stress response to the toxic elements in the growth medium [35]. The morphology of the cells shown in Figure 6 was normal and thus confirms the safety of the transferosomes. Therefore, at the concentrations evaluated, the transferosomes formulated in this study can safely be applied to ocular cells.

### 3.4. Encapsulation Efficiency and In Vitro Release Profile of CysA from Loaded Transferosomes Prepared with Tween^®^ 80

The encapsulation efficiency is a measure of the amount of drug loaded into the vesicles and represents the ability of the nanocarriers to encapsulate the enclosed drug. The encapsulation efficiency obtained from the transferosomes prepared using Tween^®^ 80 included in the hydrating fluid was 52.05% ± 2.06%, while that obtained when the Tween^®^ was included in the lipid film former before evaporation was 44.06% ± 3.01%. The edge activators were incorporated using two different approaches to delineate the effect of the incorporation method on the properties of the formed transferosomes. The EE% obtained by including the Tween^®^ 80 in the hydrating fluid was much higher than that from the alternate method. Increasing the cholesterol content, which is important for maintaining the rigidity and hence, the stability of the vesicles, has been shown to decrease the inclusion of CysA into the phospholipid membrane [36]. Additionally, CysA has been shown to partly partition between the aqueous core and the phospholipid membrane [37].

**Figure 7:**
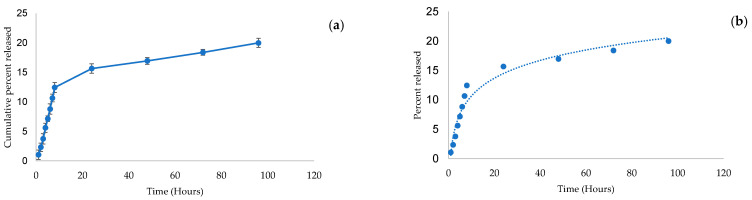
Release profile from transferosomes: (**a**) In vitro release of CysA from transferosomes; (**b**) The predicted release profile based on the Korsmeyer–Peppas model.

The in vitro release profile (Figure 7a) of the transferosomes was obtained for the Tween^®^-based formulation since it had a more favorable toxicity profile. In vitro release studies have variously been applied as an indication of the potential performance of a delivery system in vivo [38]. The release of CysA was initially rapid with over 15% of the loaded drug being released in the first 24 h. After this phase, there was a steady gradual increase over the next 72 h up to the time the release study was terminated. Other investigators have made similar observations with regards to the drug release behavior from other vesicle-based delivery systems [39]. The release data were fitted to different dissolution models and the Korsmeyer–Peppas model, which describes the release mechanism from polymeric systems, was selected as the best fit, based on the Akaike Information Criterion (AIC) and the Model Selection Criterion (MSC). The calculated Korsmeyer–Peppas constant, Kkp, was 4.106 while the n factor for the release data was 0.36, signifying that a Non-Fickian diffusion was the release mechanism from the transferosomes [35]. Figure 7b shows the predicted and the observed release data after fitting into the Korsmeyer–Peppas model.

### 3.5. Ex Vivo Corneal Permeability and Flux across Rabbits’ Cornea

**Figure 8:**
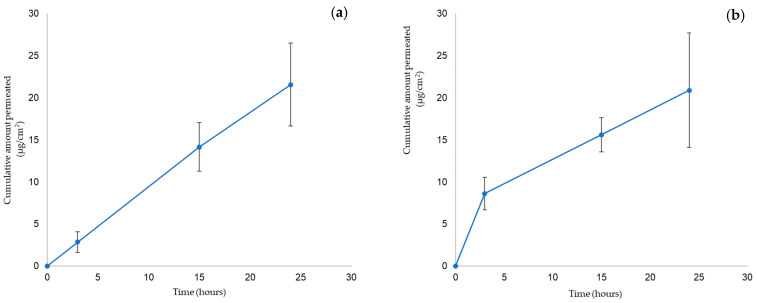
Corneal permeability of the transferosomes prepared with 1% of the edge activators (**a**) Span^®^ 80 transferosomes; (**b**) Tween^®^ 80 transferosomes.

Figure 8 shows the results of the corneal permeability studies represented as the cumulative amount of CysA diffusing per unit area plotted against time. In this study, the cumulative amount diffused per unit area increased over time during the 24 h period of investigation. The values were higher for the Tween^®^ based formulation, though the similarity factor between the permeability profiles of the two formulations was close (*F2* = 69.05). Agarwal and colleagues [40] similarly determined the permeability of CysA across the rabbit cornea, though using formulations based on semi-fluorinated alkanes. According to their study, the best formulation based on perfluorobutylpentane demonstrated a cumulative CysA permeation of 15 µg/g of the cornea over a 4-h period. Even though this is similar to the value obtained in this study for the Tween^®^ 80 formulation, the volatility of the semi-fluoroalkanes used as a solubilizer in their formulation can lead to precipitation of the dissolved drug. According to Fick’s laws of diffusion, that govern the transport of molecules across the corneal surface, the flux across the cornea depends upon the concentration gradient across the corneal barrier and the diffusion or permeability coefficient [41]. The maximum flux is usually determined from the slope of the curve for the cumulative amount permeated/unit area versus the time for the formulations not enclosed in reservoirs. The slope of the linear part of the curve for the Tween^®^- and Span^®^-based transferosome formulations were 0.78 µg/cm^2^/h and 0.912 µg/cm^2^/h, respectively, which represents the flux. The Span^®^ 80 transferosomes had a higher flux than the Tween^®^ formulation, even though the cumulative amount permeated per unit time for the Tween^®^ was initially higher. A higher flux from Span^®^ formulation indicates that, over time and at a steady state, the Span^®^ formulation will enable the permeation of higher quantities of CysA. Despite the higher flux from the Span^®^ formulation, the Tween^®^ formulation may still be preferred, considering that this difference in flux is not significant (*p* > 0.05). In addition, the Tween^®^ formulation had a more favorable toxicity profile.

## 4. Discussion

The hydrodynamic diameters obtained in this study are congruent with those observed in previous studies [42]. The linoleic acid was incorporated to replace a portion of the cholesterol as part of the lipid concentration for the membrane stabilization. Cholesterol is known to contribute to the membrane thickness and hence the increased size of the vesicles [43]. The replacement of a part of the cholesterol with linoleic acid and the CysA-loading that improves membrane fluidity all contributed to a reduction in the hydrodynamic size of the prepared transferosomes. Most of the studies report an increase in the size of the vesicles after drug loading. Shen and co-workers reported a slight decrease in the size of paclitaxel-loaded micelles, although this decrease was not consistent across all of their formulations [44]. The reduction in size observed in this study may have resulted from an effect of the CysA on the membrane packing. The defects in the lipid membrane are an important factor modulating the binding of different peripheral proteins [45], that in turn affects fluidity, and membrane thickness [46]. Pezeshky and co-workers [47] described the effect of cholesterol, for example, on the size of vesicles after 30 days of storage. The membrane thickness has been identified as one parameter that affects the size of vesicles [46]. According to Huang and co-workers, an increase in the membrane thickness leads to an increase in the vesicle size [48]. It can be inferred that CysA, being hydrophobic, is loaded into the lipid bilayer and facilitates the compact packing that reduces the thickness of the membrane, and therefore the vesicles size. The level of surfactants incorporated into ophthalmic products is critical for both the ocular cell toxicity and formulation stability. In transferosomes, an additional demand is placed on the surfactant for effective flexibility and deformability. The transferosomes enable the effective deposition of the contained drug at the site of physiological action possessing the potential to migrate through orifices smaller than their size. Tween^®^ 80 was selected because it has found use in ophthalmic products on the market due to its acceptable safety profile.

For the soy phosphatidylcholine employed in this investigation, the phase transition temperature was previously determined to be 58.1 °C in other studies [31]. Shalaev and Steponkus [49] had earlier studied the influence of sugar concentration and hydration states in DOPE-based vesicles and found that both the presence of sucrose and hydration states modified the phase transition temperature. In addition, they found that the physical state of the sugar matrix at the transition point affected the temperature of the transition and that the depression of the transition temperature for lipid vesicles always occurred if the glass transition temperature of the sugar is higher than that of the lipid used [49]. The peaks observed between 170.97 °C and 172.50 °C for the vesicles can thus be attributed to such an interdigitation of the vesicle chains. The melting endotherm for pure CysA was found at ~130 °C and the decomposition started at 226.80 °C. These peaks disappeared completely from the thermogram of the CysA-loaded transferosomes, thus indicating the efficacy and stability of the encapsulation process. Wagh and colleagues [29] made a similar observation while working with CysA nanoparticles prepared with PLGA and Eudragit 100^®^. The melting peak that appeared in the thermogram of CysA completely disappeared from the thermogram of the optimized formulation, signifying the efficacy of the encapsulation process.

The size measurements from TEM varied slightly from the DLS-determined sizes. The dehydration process may have accounted for the slight disparity in size obtained from the DLS, TEM, and SEM, as observed by Carreras and co-workers [50].

The dissolution of pure CysA was discussed and characterized in other investigations, where the poor solubility of CysA was demonstrated. For example, Dubey et al. [51] compared the drug release profiles for the CysA/micelle incorporated nanofibers with that from the corresponding quantity of pure crystalline CysA, which highlighted that there was a complete release of CysA from the nanofiber after 14 min, whereas there was negligible dissolution of the pure drug [52]. Nano-systems, such as those discussed by Dubey et al. [52] and presented herein, serve to improve the dissolution rate of the poorly soluble CysA through various mechanisms (i.e., solubilization, high surface area, improved wetting, and molecular dispersion of the drug); however, the transferosome system also serves to control the release of the drug, thus providing effective CysA levels for an extended period.

The studies that quantitate the amount of CysA released from topical formulations are limited. Most of the studies generally focus on the efficacy of CysA in the ocular condition being targeted for treatment. One study, however, evaluated the blood concentrations of CysA following the topical application of the usually administered topical doses of 0.05% and 0.1% [52]. Although the ocular concentrations of CysA were not measured, the study established that, at these doses, the 0.05% formulation was below the limit of detection while only 5.5% of the population treated with the 0.1% formulation showed detectable levels of CysA in the blood. Another study evaluated the corneal penetration of CysA from polymeric micelles in a Lewis and Brown Norway rat model [5]. The corneal CysA levels in the transplanted and healthy rats were 11710 ± 7530 ng/g and 6470 ± 1730 ng/g of tissue, respectively, and implied a superior corneal penetration performance to both a CysA oily solution and normal saline. A similar study compared a CysA micellar formulation with an emulsion formulation [23]. In their study, and over a 7-day period, 78.36% and 88.87% were released, respectively, from the emulsion and micellar formulation in vitro. The pharmacokinetic profile showed that both of the formulations displayed a similar bioavailability, but with significantly different elimination profiles, resulting in a slower elimination from the micelles. The micellar formulation was thus shown to contribute to maintaining the presence of CysA over a longer period. Even though the current transferosomal formulation presented in this investigation exhibited similar drug delivery profiles, their safety profile was improved, which was also a result of the exclusion of the toxic solvents.

The corneal epithelium has been identified as the strongest barrier limiting the passage of topically applied drugs to molecules less than 500 Da and various nano-systems have been explored to overcome this barrier. The contributions of these nano-systems towards overcoming the corneal epithelial barrier have mainly been based on the size advantage. However, evidence is also emerging to underscore the nature of the components of the nano-system viz-à-viz their interaction with the epithelial layer [7], such as interactions with the cholesterol and lipid components of the corneal epithelium, rendering it more permeable to exogenous substances [53].

## 5. Conclusions

The potential for the ocular delivery of CysA using transferosomes was explored in this investigation. The toxicity of the developed transferosomes, which is of major concern, was within acceptable limits. The transferosomes showed potential for sustaining the release of the incorporated CysA in vitro over the time period investigated in this study. The possible interaction of the loaded drug with the lipid membrane, as well as the presence of linoleic acid, may have contributed to the stabilizing effect on the size of the transferosomes over time. The corneal permeability and flux from the Span^®^ and Tween^®^ formulations were similar. A future assessment of the in vivo performance of the developed transferosomes, in terms of toxicity and therapeutic efficacy, is proposed.

## Figures and Tables

**Table 1:** Composition of transferosomes.

Component	Composition	Function
Soy Lecithin	180 mg	Lipid
Cholesterol	20 mg	Lipid
Linoleic acid	20 mg	Stabilizer/moisturizer
Tween^®^ 80	1–2% *v*/*v*	Edge activator
Span^®^ 80	1–2% *v*/*v*	Edge activator
CysA	60 mg	Active ingredient
Sucrose	2% *w*/*v*	Cryoprotectant

**Table 2:** Size distribution and polydispersity of blank and CysA-loaded transferosomes.

Parameter	Non-Lyophilized Tween^®^ 1%		Lyophilized Tween^®^ 1%		Non-Lyophilized Span^®^ 1%		Lyophilized Span^®^ 1%	
	Loaded	Blank	Loaded	Blank	Loaded	Blank	Loaded	Blank
Size (nm)	64.68 ± 0.14	69.33 ± 0.31	183.67 ± 0.62	243.01 ± 1.61	104.87 ± 0.8	159.37 ± 0.63	246.5 ± 3.09	315.7 ± 4.41
Polydispersity index	0.209 ± 0.005	0.223 ± 0.004	0.367 ± 0.01	0.388 ± 0.01	0.127 ± 0.01	0.244 ± 0.01	0.305 ± 0.02	0.388 ± 0.05
Zeta potential (mV)	−18.9 ± 1.6	−26.23 ± 1.3	−24.43 ± 2.88	−20.2 ± 2.90	−35.5 ± 1.26	−43.6 ± 4.29	−24.77 ± 0.58	−35.5 ± 3.05

**Table 3:** Effect of incorporation of linoleic acid on the stability of transferosomes.

Formulation	Size (nm)	Polydispersity Index	Zeta Potential (mV)
With Linoleic acid	175.33 ± 1.60	0.319 ± 0.32	−23.4 ± 1.00
Without Linoleic acid	200.17 ± 1.20	0.382 ± 0.01	−15.71 ± 0.17

**Table 4:** Stability profile of transferosomes.

Time (Months)	Size (nm)	Polydispersity Index	Zeta Potential (mV)
0	76.91 ± 0.81	0.504 ± 0.005	−15.93 ± 0.69
4	113.17 ± 1.11	0.277 ± 0.003	−12.7 ± 1.85

**Table 5:** Flexibility analysis of transferosomes.

Formulation	Size before Extrusion (nm)	Size after Extrusion (nm)	% Decrease in Size	Volume Loss (%)
Blank Span^®^ 80	315.77 ± 4.41	140.07 ± 2.08	55.60 ± 0.78	8 ± 1.58
CysA-loaded Span^®^ 80	246.57 ± 3.65	158.8 ± 0.59	35.72 ± 1.38	13 ± 1.05
Blank Tween^®^ 80	243.07 ± 1.61	132.77 ± 0.37	45.37 ± 0.25	6 ± 2.02
CysA-loaded Tween^®^ 80	183.17 ± 0.62	120.13 ± 0.90	34.42 ± 0.34	14 ± 1.02

## Data Availability

Not applicable.
